# Access and utilisation of leprosy healthcare services in high-burden districts in Ethiopia

**DOI:** 10.4102/sajid.v39i1.664

**Published:** 2024-12-19

**Authors:** Solomon S. Marrye, Simangele Shakwane

**Affiliations:** 1 Department of Health Studies, College of Human Sciences, University of South Africa, Pretoria, South Africa

**Keywords:** access, high-burden districts, leprosy, leprosy services, utilisation

## Abstract

**Background:**

A lack of awareness, poor quality of care, and gender inequalities are factors associated with access and utilisation of leprosy services.

**Objectives:**

This study aimed to identify factors affecting community access and utilisation of leprosy services in high-burden districts of Ethiopia.

**Method:**

A community-based cross-sectional study design was utilised and a simple random sampling technique was used to recruit study respondents. One hundred and sixty-one respondents completed the self-administered structured questionnaire. Data were analysed using SPSS version 26. A logistic regression model was used to identify predictors associated with leprosy services. A *p*-value < 0.05 was considered statistically significant.

**Results:**

More than 75% (*n* = 123) of study respondents had limited knowledge about leprosy. However, respondents who reside in urban areas were knowledgeable about the disease (adjusted odds ratio [AOR] = 8.2; 95% confidence interval [CI] = 1.6, 42.0). Men were most likely to use health care facilities (AOR [95% CI] = 2.9 [1.2, 7.2]). In addition, those who had better household income were more likely to have examined their family members for leprosy compared to low-income families (AOR [95% CI] = 4.5 [1.6, 12.9]).

**Conclusion:**

General knowledge about leprosy was low in communities. However, persons infected with leprosy who resided in the urban areas had a better understanding of leprosy. Male persons infected with leprosy were more likely to utilise leprosy services.

**Contribution:**

The results of this study provide early insights into the factors associated with leprosy service utilisation to provide community-centred leprosy care.

## Introduction

Leprosy is a disease caused by *Mycobacterium leprae,* whose natural reservoir is humans.^1^ The transmission of leprosy remains poorly understood. However, it is generally accepted that inhalation of aerosol droplets from infected individuals with high bacillary loads is the most common mechanism.^2^ Leprosy affects the skin, upper respiratory tract mucous membranes and peripheral nerves. Most people have natural immunity to leprosy, and only a few develop the disease.^2^ Leprosy is a leading cause of permanent physical disability because of infectious diseases worldwide owing to delays in diagnosis and initiation of treatment for leprosy patients.^3^

Studies have shown that several patient-related factors could influence access and utilisation of leprosy services in endemic communities.^4^ Lack of awareness about leprosy is one of the key factors behind the delay in diagnosis and initiation of treatment among leprosy sufferers. Therefore, leprosy awareness activities advocate the detection of leprosy at an early stage of disease development.^5^ Furthermore, the infected communities need to be informed that leprosy can be easily treated and cured through the involvement of patients, including their family members and affiliated associations such as persons infected with leprosy.^4^

Most people infected with leprosy practice self-medication immediately after the onset of symptoms, and therefore, they tend to rely on home-based remedies.^5^ In contrast, poor quality of health care can delay the diagnosis of leprosy early in disease development in leprosy-endemic areas.^6^ The risk of misdiagnosis for leprosy patients who received substandard health care is higher than for leprosy patients who received better health care. In addition, people infected with leprosy have been stigmatised and discriminated against by the attitudes and beliefs of society towards the disease because of misconceptions about the lack of treatment and visible deformities because of nerve damage.^7^ In particular, women infected with leprosy are more likely to be underreported to the national programmes and less likely to be detected at the health care facilities because of associated factors such as low socio-economic status, limited mobility, illiteracy and low levels of leprosy knowledge and cultural influences.^8^

Other studies have also mentioned that health system-related factors such as health service organisation and health worker skills may influence the accessibility and utilisation of leprosy treatments.^9^ A study conducted in China shows that the provision of leprosy contact tracing services from the registered index cases of leprosy was only 60%.^10^ Therefore, assessing the accessibility of leprosy care can play an important role in improving coverage and smooth service delivery in leprosy-infected communities.^11^ However, few studies have been conducted to assess factors that may influence leprosy service utilisation among infected communities in high-burden districts in Ethiopia. Therefore, this study aimed to identify the factors that influence leprosy service utilisation among communities in high-burden districts of Ethiopia. Leprosy high-burden districts are districts with average 6 years’ leprosy prevalence rate accounting for ≥ 1 case per population of 10 000.^12^ The majority of high-burden districts (91.4%) were found in Amhara and Oromia regions, contributing 54% of all leprosy cases notification to the national programme.^12^ For example, 2046 leprosy cases were reported from the Oromia region, while 1409 cases were reported from the Amhara region in 2018.^13^ Moreover, the World Health Organization (WHO)’s report for the year 2023 considered Ethiopia as one of the top 23 global leprosy priority countries, which notified 2966 new leprosy cases in 2022.^14^

## Research methods and design

The authors declare that this research paper was part of a larger community-based intervention research design study that focused on Phase 1 of the research – Information gathering and synthesis. The theoretical framework of this research was based on the Theory of Change of Model (ToCM). Theory of Change of Model is a procedural production of the programme to describe the list of activities that lead to the desired outcomes and the connections between programme activities and outcomes at each stage.^15^ Moreover, ToCM identifies gaps or opportunities for improvement.^16^

### Study design

A cross-sectional study design was used to determine the leprosy service utilisation and associated factors among leprosy-infected individuals in leprosy-burdened districts of Ethiopia. Cross-sectional research design is the collection of data at a specific point in time to describe the status of relationships between phenomena.^17^ Therefore, this cross-sectional study was applied to describe the use and identification of factors associated with leprosy diagnosis, treatment and contact tracing services.

### Study setting

The study was conducted in two selected leprosy high-burden districts of the Amhara and Oromia regions of Ethiopia.

### Study population

The population for this phase of the study consisted of individuals who had been diagnosed and treated in the health care facilities of leprosy high-burden districts in the two selected districts of Ethiopia in the last 5 years.

### Sampling technique and procedure

Using a simple random sampling technique, leprosy-infected individuals who had been diagnosed and treated were selected from the register lists of the health care facilities in high-burden districts. The contact details of the persons infected with leprosy were obtained from the leprosy registration books. The health extension workers employed at the community health posts were tasked with tracing the leprosy patients living in their catchment areas. Leprosy patients were then gathered at healthcare facilities, and information was given about the nature of the study. Finally, those persons infected with leprosy who could consent to participate in the study were sampled. The exclusion criteria were those who had not been diagnosed in high leprosy districts’ health care facilities and whose leprosy documents were not found for the last 5 years.

### Sample size

The required sample size was determined using a single proportion formula (Equation 1):


$$n=\frac{{\left(Z_{\propto /2}\right)}^{2}p(1-p)}{\Delta^{2}}$$ [Eqn 1]

Where

*n* = Sample size.

*Z* = The *Z* score of confidence interval (CI) at 95% takes the standard value of 1.96.

*P* = The prevalence of leprosy in high-burden districts.

Δ = The margin of error (5%) between the population and sample.

Therefore, considering the estimated prevalence (5%) of leprosy in high-burden districts,^12^ the sample size was calculated to be 73. As this study used two districts, the overall sample size was 146. Moreover, by the assumption of the missed patients in the registration books in the health care facilities, this study estimated 10% of the non-respondent rate. Therefore, the overall sample size was calculated as 161.

### Data collection and procedure

A self-administered questionnaire was used to collect the necessary information. The self-administered questionnaires were initially prepared in English and translated into local languages such as Amharic and Oromifa. The data collection tool collected information on factors associated with delay in diagnosis and treatment, including contact tracing from the perspective of persons infected with leprosy. The first part of the questions dealt with the socio-demographic characteristics of the respondents, and the second part focused on patients’ understanding of the leprosy problems, diagnosis and treatment of leprosy. The second part of the questions also discussed factors determining delays in leprosy contact tracing activities, diagnosis and treatment linkage services.

Two data collectors were trained in data collection procedures and ethical considerations. Data were collected in the health care facilities through maintaining COVID-19 pandemic regulations during Phase 1 of the research (between January and June 2022).

### Variables

The dependent variables were associated with the leprosy services such as leprosy diagnosis, contact tracing and treatment linkage activities. The independent variables were factors that affect the utilisation of leprosy services by persons infected with leprosy.

### Data management and analysis

Data cleaning was performed through manual review using SPSS version 26 software packages in the early stage of data collection and entry processes. Questionnaires that were not completed and lacked consistency were discarded. Furthermore, after data entry, the consistency of all data was ensured using the software SPSS version 26.0 packages by performing a simple frequency, split and select analysis.

Logistic regression analysis was performed in response to the research objectives and associated variables (dependent and independent variables). Moreover, data were entered and summarised using frequencies and percentages. *P*-value < 0.05 was considered statistically significant to determine the degree of association among the study variables. The mean value of the variables was used to report the findings within its 95% CI.

### Data validity

In this study, the self-administered questionnaires were pre-tested to measure what they were supposed to measure by involving 30 respondents from persons infected with leprosy. The questionnaires were modified for the questions the respondents found challenging to understand. The data obtained during pre-testing were not considered part of the main study.

### Data reliability

In this study, to ensure the reliability of the data collection tools, a Cronbach’s alpha (α) test was applied. This test is a coefficient that tests or tells about the internal consistency of a given measurement tool.^18^ The coefficient of α is a value that ranges between 0.0 and 1.0. A score of ≥ 0.7 will imply that the instrument is acceptable or reliable. Those with a score of less than 0.7 will denote that there will be no consistency in measurement.^18^ Therefore, Cronbach’s α test value was found to be 0.7 for the person infected with leprosy self-administered questionnaires.

### Ethical considerations

Data were collected after obtaining an Ethical Clearance Certificate from the College of Human Sciences Research Ethics Review Committee (CREC) at the University of South Africa with CREC reference no: 2020-CHS-67128815. In addition, approval letters were received from Amhara and Oromia Regional Health Bureau Ethical Clearance Committees with reference number N/o/H/R/T/T/D/5/8 and BEFO/U/BTFH/H6/108, respectively. Written informed consent was obtained from respondents before data collection. The respondents were informed of their full right to decline participation in the study during the data collection process.

## Results

### Socio-demographic characteristics

According to the calculated sample size, a total of 161 persons infected with leprosy were enrolled, and all respondents were literate in this study phase; the response rate was 100%. Of the total number of respondents, 102 (63.4%) were men. Forty-eight (29.8%) of the study respondents were between 30 and 39 years. The average mean age of the study respondents was 38.9 (standard deviation [s.d.] = ±15.2) years. The respondents’ age range was between 18 and 65 years, and the median age was 48 (29.8%) being between 30 and 39 years. One hundred fifty (90%) of the respondents were rural residents. Accordingly, 81 (50.3%) of the respondents were unemployed, 57 (35.4%) were retired and 23 (14.3%) were self-employed. Regarding education level and household income, 111 (68.9%) of the respondents had never attended education and 91 (56.5%) had a monthly household income of less than 500 Birrs (see Table 1).

**TABLE 1:** Socio-demographic characteristics of persons infected with leprosy in leprosy high-burden districts of Amhara and Oromia regions (*n* = 161).

Variables	*n*	%
**Age (years)**		
18–29	43	26.7
30–39	48	29.8
40–49	31	19.3
50–59	18	11.2
> 60	21	13.0
**Gender**		
Male	102	63.4
Female	59	36.6
**Place of residence**		
Urban	11	6.8
Rural	150	93.2
**Employment status**		
Unemployed	81	50.3
Self-employed	23	14.3
Retired	57	35.4
**Level of education**		
Never attended formal education	111	68.9
Primary school	36	22.4
Secondary school	11	6.8
Higher education	3	1.9
**Household income in birr†**		
> 3500	8	5.0
3500–1500	11	6.8
1500–500	51	31.7
< 500	91	56.5

Note: Please see the full reference list of this article for details on the cited article: Marrye SS, Shakwane S. Access and utilisation of leprosy healthcare services in high-burden districts in Ethiopia. S Afr J Infect Dis. 2024;39(1), a664. https://doi.org/10.4102/sajid.v39i1.664.

† , The food poverty line and poverty line (total poverty line) have been calculated to be 400 and 586 birrs per adult per month in Ethiopia.^19^

### Knowledge of leprosy and its services

The knowledge of leprosy by persons infected with leprosy was classified as high if they were able to provide correct responses to at least three of four questions (≥ 75%) describing the nature of the disease (i.e. it is airborne; it affects skin, nerve and the inner side of the respiratory tract; it is curable and it causes disability if it has not been treated early). The number of respondents who showed a high level of knowledge was 38 (23.6%), while most respondents (123 [76.4%]) scored below 75% correct responses.

Of the persons infected with leprosy (*n* = 161) in the study, 100 (62.1%) had heard about leprosy before they were diagnosed with the disease themselves. Among the respondents who had heard about leprosy, 72 (44.7%) had accurately responded that leprosy could be transmitted through airborne means from infected patients. Moreover, 46 (28.6%) of the study respondents responded that leprosy is a curable disease, and 42 (26.1%) mentioned that leprosy causes disability if it is not treated early. However, 30 (18.6%) thought that leprosy can be inherited (see Table 2).

**TABLE 2:** Knowledge of leprosy and access to care by the persons infected with leprosy in high-burden districts of Amhara and Oromia regions.

Variables	*n*	%
**Knowledge of leprosy**		
Hereditary disease	30	18.6
Transmitted through airborne means	72	44.7
Affects skin, nerve and inner side of the respiratory tract	25	15.5
Curable disease	46	28.6
Causes disability if it has not been treated early	42	26.1
**Symptoms of persons infected with leprosy**		
Pale patches on your skin	133	82.6
Lumpy or thickened skin	58	36.0
Pain or tingling in your arms 16	9.9	-
Loss of feeling on patches of skin or hands or feet	53	32.9
Muscle weakness in your hands feet, arms or legs	35	21.7
Muscle weakness around your eyes	21	13.0
Cuts, wounds or ulcers	25	15.5
**Diagnosis by healthcare practitioners at first visit**		
Yes	35	21.7
No	126	78.3
**Reasons not to visit healthcare practitioners**		
Fear of being identified	124	77.0
Unavailability of health care facilities	20	12.4
Quality of services provided	16	9.9
Influence of the traditional leader	30	18.6
**Examination of leprosy of family members**		
Yes	49	30.4
No	112	69.6
**Reasons family members were not examined**		
Lack of information about the diseases	92	57.2
Fear of stigma by the community	86	53.4
Health professionals do not instruct me	94	58.4
**Referral to another health facility by the health care practitioner**		
Yes	64	39.8
No	97	60.2
**Distance from the closest health care facility (km)**		
0–10	83	51.6
10–20	44	27.3
> 20	34	21.1
**Waiting time for diagnosis of leprosy (weeks)**		
0–12	123	76.4
12–24	18	11.2
24–52	13	8.1
> 52	7	4.3

Note: Numbers may not add up to 100% as multiple responses are possible.

### Factors affecting the knowledge of leprosy

A multivariate logistic regression analysis was used to determine factors affecting the knowledge of persons infected with leprosy. In multivariate logistic regression analysis, variables such as age group of 50 to 59 years, urban residents and self-employed showed significant association with knowledge of leprosy (*p* < 0.05). Persons infected with leprosy within the age group of 50 to 59 years had less understanding of leprosy compared to the younger age groups (adjusted odds ratio [AOR] [95% CI] = 0.04 [0.003, 0.8]). However, persons infected with leprosy who reside in urban areas were more knowledgeable about leprosy than those who live in rural areas (AOR [95% CI] = 8.2 [1.6, 42.0]). Moreover, employed persons infected with leprosy were also more knowledgeable than unemployed persons (AOR [95% CI] = 21.3 [3.9, 117]) (see Table 3).

**TABLE 3:** Factors affecting knowledge of leprosy among persons infected with leprosy in high-leprosy-burdened districts of Amhara and Oromia regions.

Variables	Knowledge of leprosy				COR	95% CI	AOR	95% CI
	High		Low					
	*n*	%	*n*	%				
**Age**								
18–29	13	8.1	30	18.6	Reference	Reference	Reference	Reference
30–39	13	8.1	35	21.7	0.9	0.3, 2.1	0.5	0.2, 1.6
40–49	6	3.7	25	15.5	0.6	0.2, 1.7	0.3	0.1, 1.1
50–59	1	0.6	17	10.6	0.04	0.02, 1.10	0.04	0.003, 0.800*
> 60	5	3.1	16	9.9	0.72	0. 2, 2.4	0.8	0.21, 3.28
**Gender**								
Male	26	16.2	76	47.2	1.3	0.6, 2.9	1.6	0.6, 4.2
Female	12	7.5	47	29.2	Reference	Reference	Reference	Reference
**Place of resident**								
Urban	7	4.4	4	2.5	6.7	1.8, 24.4**	8.2	1.6, 42.0*
Rural	31	19.3	119	73.9	Reference	Reference	Reference	Reference
**Employment status**								
Unemployed	16	9.9	65	40.4	1.5	0.6, 3.8	2.1	0.7, 6.0
Self-employed	14	8.7	9	5.6	9.0	3.1, 29.3**	21.3	3.9, 117.0**
Retired	8	5.0	49	30.4	Reference	Reference	Reference	Reference
**Level of education**								
Never studied	21	13.0	90	56.0	Reference	Reference	Reference	Reference
Primary school	12	7.5	24	14.9	2.1	0.9, 5.0	1.0	0.3, 3.4
Secondary school	3	1.9	8	5.0	1.6	0.4, 6.6	0.3	0.03, 4.80
Higher education	2	1.2	1	0.6	8.6	0.7, 99.0	2.5	0.03, 171.80
**Household income in birr†**								
< 500	20	12.4	71	44.1	Reference	Reference	Reference	Reference
1500–500	10	6.2	41	25.5	0.9	0.4, 2.0	1.13	0.4, 3.2
3500–1500	5	3.1	6	3.7	3.0	0.81, 10.70	3.6	0.5, 27.9
> 3500	3	1.2	5	3.1	2.1	0.5, 9.7	0.4	0.01, 11.10

AOR, adjusted odds ratio; CI, confidence interval; COR, crude odds ratio.

* , *p* < 0.05 indicate significant association;

** , *p* < 0.001 indicate strong significant association.

† , The food poverty line and poverty line (total poverty line) have been calculated to be 400 and 586 birrs per adult per month in Ethiopia.^19^

### Factors affecting the utilisation of leprosy diagnosis and treatment services

More than 126 (75%) persons infected with leprosy did not get a diagnosis and/or treatment services during the first visit to the health care facilities. Sixty-four (39.8%) persons infected with leprosy were referred to other health care facilities for further diagnosis and treatment services. At the same time, 83 (51.6%) persons infected with leprosy could access the health care facilities in the nearby area (0 km – 10 km). Furthermore, 123 (76.4%) persons had an average waiting time of up to 12 weeks during the first diagnosis of leprosy.

In multivariate logistic regression analysis, variables such as gender and waiting time to the first diagnosis of leprosy showed a significant association with health care facility use for leprosy diagnosis and treatment services. The male persons infected with leprosy were most likely to use health care facilities at the time of illness compared to female persons (AOR [95% CI] = 2.9 [1.2, 7.2]). However, the persons infected with leprosy who had a waiting time of 12–24 weeks from the start of illness were less likely to use the health care facility (AOR [95% CI] = 0.1 [0.03, 0.5]).

Additionally, in bivariate logistic regression analysis, variables such as being employed and availability within a 10 km radius of the health care facility showed significant association with health care facility use. For example, the persons infected with leprosy who are employed had a better chance of utilising health care facilities (COR [Crude odds ratio] [95% CI] = 4.7 [1.02, 21.5]) compared to those unemployed. Likewise, the persons infected with leprosy who live within a 10 km radius of the health care facility were more likely to utilise the leprosy diagnosis and treatment services (COR [95% CI] = 3.2 [1.3, 8.2]) (see Table 4).

**TABLE 4:** Factors affecting the utilisation of leprosy diagnosis and treatment services among persons infected with leprosy in high-leprosy-burdened districts of Amhara and Oromia regions.

Variables	Health care facility use				COR	95% CI	AOR	95% CI
	Yes		No					
	*n*	%	*n*	%				
**Age (years)**								
18–29	36	22.4	7	4.4	2.1	0.6, 7.2	-	-
30–39	40	24.8	8	5.0	2.0	0.6, 6.7	-	-
40–49	22	13.7	9	5.6	1.0	0.3, 3.3	-	-
50–59	13	8.1	5	3.1	1.0	0.3, 4.2	-	-
> 60	15	9.3	6	3.7	Reference	Reference	-	-
**Gender**								
Male	87	54.0	15	9.3	2.9	1.4, 6.4**	2.9	1.2, 7.2*
Female	39	24.2	20	12.4	Reference	Reference	Reference	Reference
**Place of resident**								
Urban	10	6.2	1	0.6	2.9	0.4, 23.7	-	-
Rural	116	72.1	34	21.1	Reference	Reference	-	-
**Employment status**								
Unemployed	56	34.8	25	15.5	Reference	Reference	Reference	Reference
Self-employed	21	13.0	2	1.2	4.7	1.02, 21.50*	13.9	0.97, 200.00
Retired	49	30.4	8	5.0	2.7	1.13, 6.60*	2.4	0.8, 6.9
**Level of education**								
Never studied	81	50.3	30	18.6	Reference	Reference	Reference	Reference
Primary school	32	19.9	4	2.5	2.9	1.0, 9.2	1.1	0.31, 4.10
Secondary school	11	6.8	-	-	-	-	-	-
Higher education	2	1.2	1	0.6	0.7	0.1, 8.5	0.03	0.0006, 1.4800
**Household income in birr†**								
< 500	67	41.6	24	14.9	Reference	Reference	Reference	Reference
1500–500	42	26.1	9	5.6	1.7	0.7, 3.9	0.9	0.3, 2.5
3500–1500	10	6.2	1	0.6	3.6	0.4, 29.5	2.9	0.1, 59.6
> 3500	7	4.4	1	0.6	2.5	0.3, 21.5	-	-
**Distance from the nearby health care facility (km)**								
0–10	71	44.1	12	7.5	3.2	1.3, 8.2*	2.1	0.7, 6.6
10–20	33	20.5	11	6.8	1.6	0.6, 4.4	1.3	0.4, 4.2
> 20	22	13.7	12	7.5	Reference	Reference	Reference	Reference
**Waiting time to the first diagnosis of leprosy (weeks)**								
0–12	103	64.0	20	12.4	Reference	Reference	Reference	Reference
12–24	10	6.2	8	5.0	0.2	0.1, 0.7*	0.1	0.03, 0.50*
24–52	9	6.0	4	2.5	0.4	0.1, 1.6	0.6	0.1, 2.7
> 52	4	2.5	3	1.9	0.3	0.05, 1.20	0.1	0.01, 1.00

Note: Please see the full reference list of this article for details on the cited article: Marrye SS, Shakwane S. Access and utilisation of leprosy healthcare services in high-burden districts in Ethiopia. S Afr J Infect Dis. 2024;39(1), a664. https://doi.org/10.4102/sajid.v39i1.664.

AOR, adjusted odds ratio; CI, confidence interval; COR, crude odds ratio.

* , *p* < 0.05 indicate significant association;

** , *p* < 0.001 indicate strong significant association.

† , The food poverty line and poverty line (total poverty line) have been calculated to be 400 and 586 birrs per adult per month in Ethiopia.^19^

### Factors affecting the utilisation of leprosy contact tracing services

One hundred twelve (70%) family members of persons infected with leprosy were not traced and examined for active signs of leprosy (see Table 2). Out of 38 (23.6%) (*n* = 161) persons infected with leprosy who had an active sign of leprosy, only 19 (50%) persons were offered leprosy contact tracing services for their family members.

In multivariable logistic regression model analysis, a variable such as the monthly household income of 1500–500 birr showed a significant association with tracing leprosy contacts among persons infected with leprosy (*p* < 0.05). Furthermore, the respondents whose household income was within the range of 1500–500 birr were more likely to have examined their family members for leprosy compared to those below income (AOR [95% CI] = 4.5 [1.6, 12.9]).

Moreover, in bivariate logistic regression analysis, variables such as being self-employed, having a secondary level of education and having a monthly household income of more than 1500 birr were significantly associated with receiving the service of leprosy contact tracing from health care practitioners (*p* < 0.05). The poverty line for Ethiopia is < 500 birr. Moreover, the persons infected with leprosy who are self-employed were more likely to have examined their family members at the health care facilities (COR [95% CI] = 2.7 [1.03, 7.2]).

Likewise, the persons infected with leprosy who have a secondary level of education had examined their family members for leprosy (COR [95% CI] = 29 [3.5, 242]) compared to those who had never attended education. The persons infected with leprosy whose household income was more than 1500 birr were more likely to have traced their family members for contact with leprosy by health care practitioners (see Table 5).

**TABLE 5:** Factors affecting tracing of leprosy contacts among persons infected with leprosy in high-leprosy-burden districts of Amhara and Oromia regions.

Variables	Tracing of leprosy contacts				COR	95% CI	AOR	95% CI
	Yes		No					
	*n*	%	*n*	%				
**Age (years)**								
18–29	11	6.8	32	20	Reference	Reference	-	-
30–39	17	10.6	31	19.3	1.6	0.6, 3.9	-	-
40–49	9	5.6	22	13.7	1.2	0.4, 3.3	-	-
50–59	7	4.4	11	6.8	1.9	0.6, 6.0	-	-
> 60	5	3.1	16	9.9	0.9	0.3, 3.1	-	-
**Gender**								
Male	28	17.4	74	46.0	Reference	Reference	-	-
Female	21	13.0	38	23.6	1.5	0.7, 2.9	-	-
**Place of resident**								
Urban	-	-	11	6.8	-	-	-	-
Rural	49	30.4	101	62.7	-	-	-	-
**Employment status**								
Unemployed	33	20.5	48	29.8	Reference	Reference	-	-
Self-employed	15	9.3	8	5.0	2.7	1.03, 7.20**	0.55	0.12, 2.80
Retired	1	0.6	56	34.8	0.03	0.003, 0.200**	Reference	Reference
**Level of education**								
Never studied	28	17.4	83	51.6	Reference	Reference	Reference	Reference
Primary school	10	6.2	26	16.2	1.1	0.5, 2.6	1.5	0.4, 5.4
Secondary school	10	6.2	1	0.6	29	3.5, 242.0*	-	-
Higher education	1	0.6	2	1.2	1.5	0.1, 17.0	-	-
**Household income in birr†**								
< 500	18	11.2	73	45.3	Reference	Reference	Reference	Reference
1500–500	16	9.9	35	21.7	1.8	0.9, 4.1	4.5	1.6, 12.9*
3500–1500	9	5.6	2	1.2	18	3.6, 91*	-	-
> 3500	6	3.7	2	1.2	12	2.3, 65.0*	-	-
**Distance from the nearby health care facility (km)**								
0–10	22	13.7	61	37.9	Reference	Reference	Reference	Reference
10–20	14	8.7	30	18.6	1.3	0.6, 2.8	0.6	0.2, 1.9
> 20	13	8.1	21	13	1.7	0.7, 4.0	1.0	0.3, 3.1
**Waiting time to first diagnosis of leprosy (weeks)**								
0–12	37	23	86	53.4	Reference	Reference	-	-
12–24	7	4.4	11	6.8	1.5	0.5, 4.1	-	-
24–52	4	2.5	9	5.6	1.03	0.3, 3.6	-	-
> 52	1	0.6	6	3.7	0.4	0.1, 3.3	-	-

Note: Please see the full reference list of this article for details on the cited article: Marrye SS, Shakwane S. Access and utilisation of leprosy healthcare services in high-burden districts in Ethiopia. S Afr J Infect Dis. 2024;39(1), a664. https://doi.org/10.4102/sajid.v39i1.664.

AOR, adjusted odds ratio; CI, confidence interval; COR, crude odds ratio.

* , *p* < 0.05 indicate significant association;

** , *p* < 0.001 indicate strong significant association.

† , The food poverty line and poverty line (total poverty line) have been calculated to be 400 and 586 birrs per adult per month in Ethiopia.^19^

## Discussion

This study aimed to identify factors affecting community access and utilisation of leprosy services in leprosy high-burden districts in Ethiopia. The findings suggest that overall knowledge about leprosy was low among persons infected with leprosy. Those infected with leprosy did not use the leprosy diagnosis and treatment services as expected.

This study showed that leprosy-infected individuals living in urban areas were more knowledgeable compared to those in rural areas (AOR [95% CI] = 8.2 [1.6, 42.0]). The study conducted in the eastern part of Ethiopia indicates that being an urban inhabitant was significantly associated with having a high level of knowledge of leprosy.^20^ Studies in India also revealed that most of the urban residents in the district of Tamil Nadu were knowledgeable about leprosy.^21^ Furthermore, infected communities need to be informed that leprosy can be treated and cured through the participation of patients, including their family members and affiliated associations such as persons infected with leprosy.^3^ Unfortunately, persons infected with leprosy from rural areas lacked the necessary knowledge about their disease, leading to poor treatment linkage.

The findings of this study also showed that male persons infected with leprosy were more likely to use health care facilities at the time of illness compared to female persons (AOR [95% CI] = 2.9 [1.2, 7.2]). These results are supported by the study that reflected contributing factors for the resurgence of leprosy in Timor.^22^ A study done in Brazil also revealed that most leprosy patients diagnosed at health care facilities were male persons.^23^ Results indicated that female persons infected with leprosy did not have a chance to access and use the leprosy services in the nearby health care facilities because of social stigma, low socio-economic conditions and other cultural issues such as gender inequalities.^4^

This study found that persons infected with leprosy who are employed had a better chance of utilising diagnostic and treatment services than unemployed people (COR [95% CI] = 4.7 [1.02, 21.5]). Higher income facilitates access to leprosy diagnosis and treatment and is associated with better leprosy treatment adherence and outcomes.^24^ Therefore, focusing on providing early detection and treatment services to lower-income groups and focusing on women as well could make a significant contribution to national leprosy control.^25^

The study revealed that persons infected with leprosy who have a secondary level of education were more likely to have their family members traced and examined for active signs of leprosy compared to those who had never attended education (COR [95% CI] = 29 [3.5, 242]). A study done in Benin on factors associated with leprosy screening activities indicated that a delay in screening family members of the first index is significantly associated with having education.^26^

This study also signifies that the persons infected with leprosy whose household had better income were more likely to have traced and screened their family members for active signs of leprosy (COR [95% CI] = 18 [3.6, 91]). There is a connection between the improved use of leprosy contact tracing services and a higher proportion of households with better socio-economic conditions.^27^ Therefore, the economic condition of the family has a great influence on the patient’s leprosy care-seeking behaviour, especially on diagnosis, treatment and contact tracing services.^28^ This means that families with a better household income can seek medical treatment at the onset of leprosy, improving health outcomes.

Overall, the study assessed and identified the gaps related to leprosy service use in high-burden districts. Socioe-conomic, geographical location and being women were identified as influencing factors in leprosy service utilisation in communities in high-burden districts in Ethiopia. Therefore, leprosy services should focus on rural areas, poor communities and women as persons from these groups lack knowledge about leprosy and are stigmatised because of their status in society. The results of this study strongly advocate the importance of designing and implementing new community-based interventions to strengthen leprosy control activities such as early case notification, treatment linkage and contact tracing, making leprosy health services inclusive and accessible to all persons infected with leprosy.

### Limitations of the study

This phase of the study used a quantitative cross-sectional design, and the experiences of leprosy-infected individuals were not explored. Data were collected during political unrest in some parts of Ethiopia and COVID-19 pandemic restrictions limiting data collection locations and contact with leprosy-infected persons. Persons infected with leprosy are a vulnerable group as they are sometimes excluded from society and even families. Future studies should include illiterate people, children and women to eliminate stigma and leprosy-related disability.

## Conclusion

This study showed that people infected with leprosy had little knowledge about leprosy and its treatment. Gender, household income, level of education and employment status were some of the factors that influenced access and utilisation of leprosy services in high-burden districts in Ethiopia. Therefore, it is important to design and periodically develop campaigns to raise awareness activities considering those factors identified in the study to improve leprosy service utilisation and to improve awareness of the disease by staff of health care facilities.
